# Supplementary material for: ABaCo: addressing heterogeneity challenges in metagenomic data integration with adversarial generative models
Source: Nucleic Acids Res. 2026 Mar 17;54(5):gkag227. doi: 10.1093/nar/gkag227 (PMC12993454; doi:10.1093/nar/gkag227)
Supplement: gkag227_Supplemental_Files [file gkag227_supplemental_files.zip › Supplementary_File_1_revised.pdf]

Supplementary Material

Tables and Figures

Dataset	Epochs (1st, 2nd, 3rd)	Phases lr. (1st, 2nd, 3rd)	Disc. lr.	Adv. lr.
Anaerobic digestion	2000<br>2000<br>1000	1e-3<br>1e-3<br>1e-6	1e−3	1e−3
Inflammatory Bowel Disease	4000<br>1000<br>2000	1e-4<br>1e-6<br>1e-6	1e−6	1e−6
DTU-GE sewage	4000<br>1000<br>1000	2e-4<br>1e-6<br>1e-7	1e−6	1e−6
Simulated data Batch effect only	2000<br>2000<br>1000	1e-4<br>1e-5<br>1e-7	1e−5	1e−5
Simulated data Batch and biological effect	5000<br>2000<br>1000	2e-4<br>1e-6<br>1e-6	1e−7	1e−7

Supplementary Table 1: Training schedule for simulated and case study datasets. The table lists the number of epochs for each training phase (Phase 1–3), the VAE learning rate in each phase (Phase lr.), the batch-discriminator learning rate (Disc. lr.), and the encoder adversarial learning rate used during discriminator updates (Adv. lr.).

Dataset	Bio. penalty	Clust. penalty	NLL	KL-div.
Anaerobic digestion	1.0	1.0	1.0	1.0
Inflammatory Bowel Disease	1e-2	1.0	1e-3	1e-3
DTU-GE sewage	100.0	1.0	1.0	1.0
Simulated data Batch effect only	0.0	0.0	1.0	1.0
Simulated data Batch and biological effect	10.0	10.0	1.0	10.0

Supplementary Table 2: Weights used for the loss function in simulated and case study datasets. The table shows the weight of the biological preservation penalty (Bio. penalty), the cluster overlap penalty (Clust. penalty), the negative log-likelihood (NLL), and the KL-divergence (KL-div.) terms of the ELBO.

Dataset	Samples	Taxa	Decoder	Compute Metrics		
				CPU Time (s)	Single GPU Util. (%)	Peak GPU Mem. (GiB)
Anaerobic Digestion	75	567	ZINB<br>NB	77.2<br>62.8	26<br>24	0.07<br>0.06
Inflammatory Bowel Disease	524	435	ZINB<br>NB	117.1<br>115.3	19<br>17	0.05<br>0.05
DTU-GE sewage	129	162	ZINB<br>NB	112.9<br>103.7	24<br>22	0.04<br>0.04
Simulated data: Batch effect only	200	1000	ZINB<br>NB	75.7<br>71.9	27<br>24	0.10<br>0.08
Simulated data: Batch and biological effect	200	1000	ZINB<br>NB	121.1<br>109.2	27<br>25	0.10<br>0.08

Supplementary Table 3: Compute performance of ABaCo with different decoder output distributions. **ZINB**: Zero-inflated Negative Binomial; **NB**: Negative Binomial.

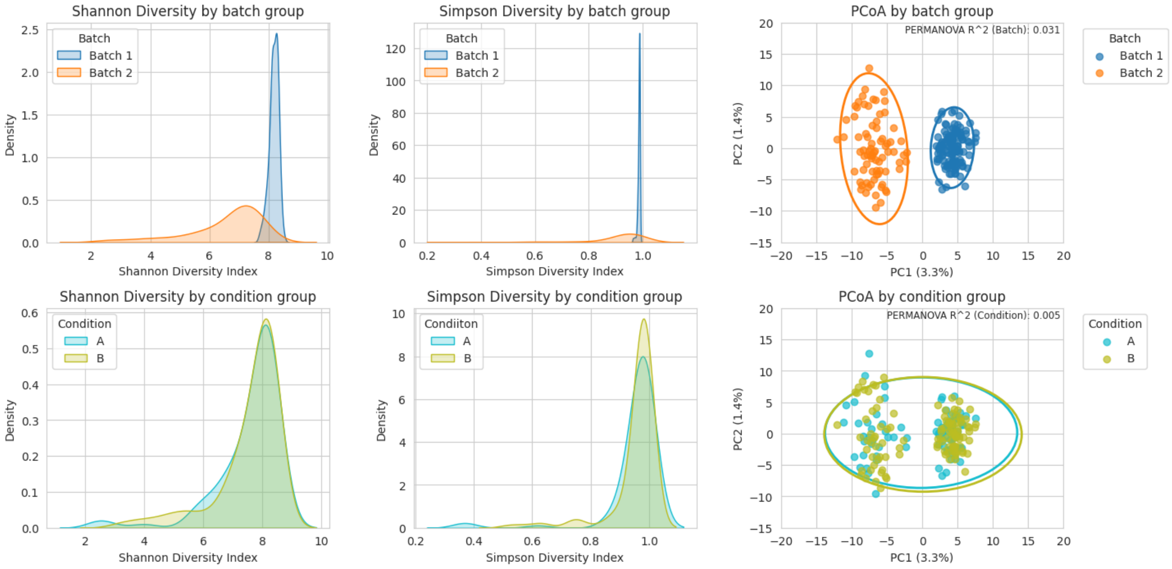

Supplementary Figure 1: Kernel density estimates of Shannon and Simpson alpha diversity (left, center) and PCoA on Aitchison distances (right), shown by batch (top) and biological group (bottom) for the simulated dataset with batch effect only.

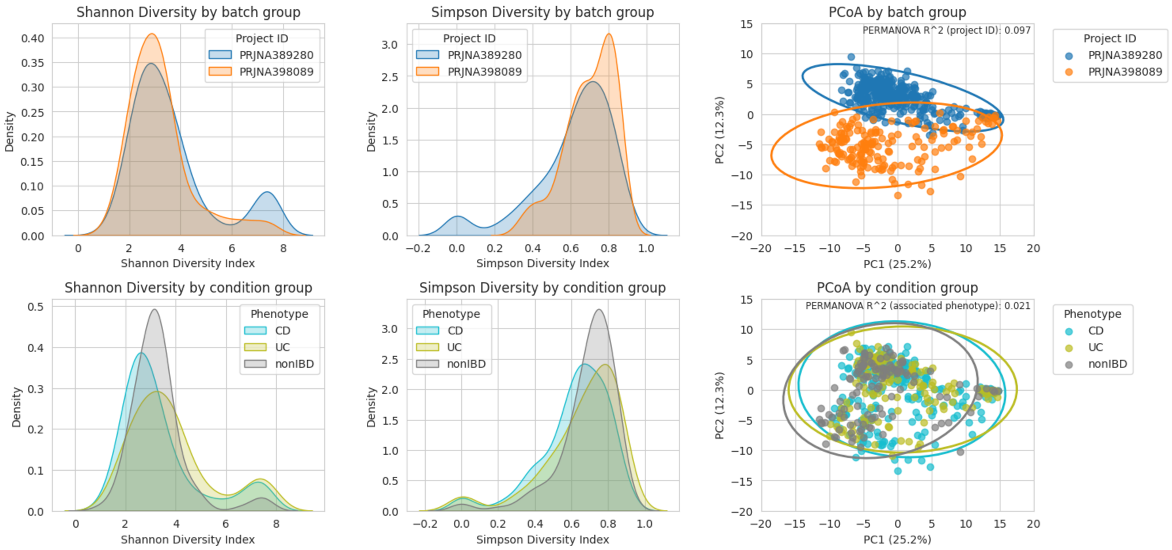

Supplementary Figure 2: Kernel density estimates of Shannon and Simpson alpha diversity (left, center) and PCoA on Aitchison distances (right), shown by batch (top) and biological group (bottom) for the IBD case study dataset.

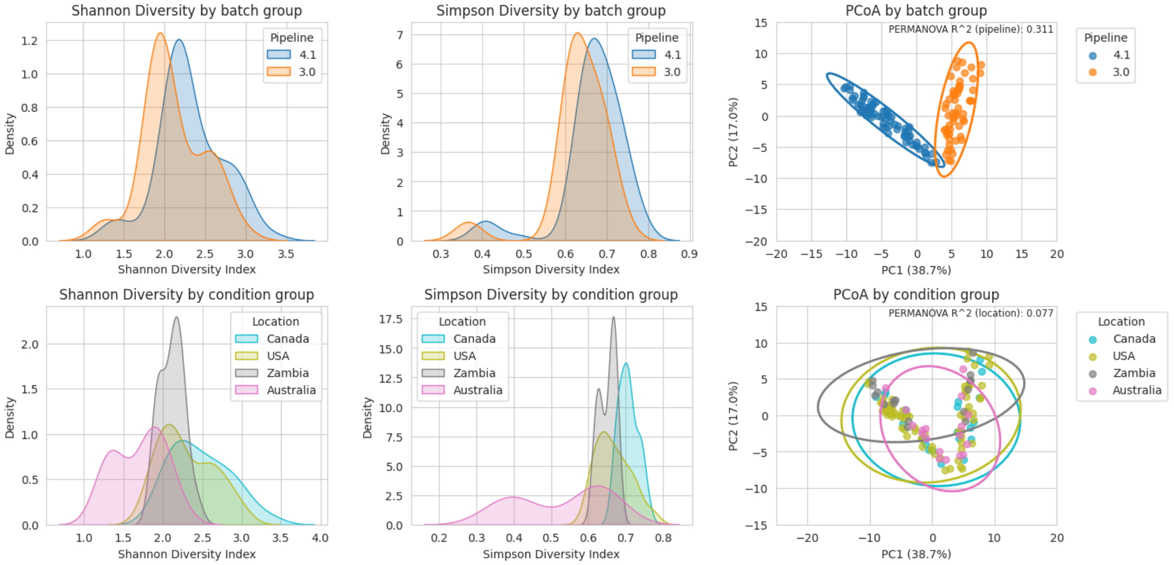

Supplementary Figure 3: Kernel density estimates of Shannon and Simpson alpha diversity (left, center) and PCoA on Aitchison distances (right), shown by batch (top) and biological group (bottom) for the DTU-GE sewage case study dataset.

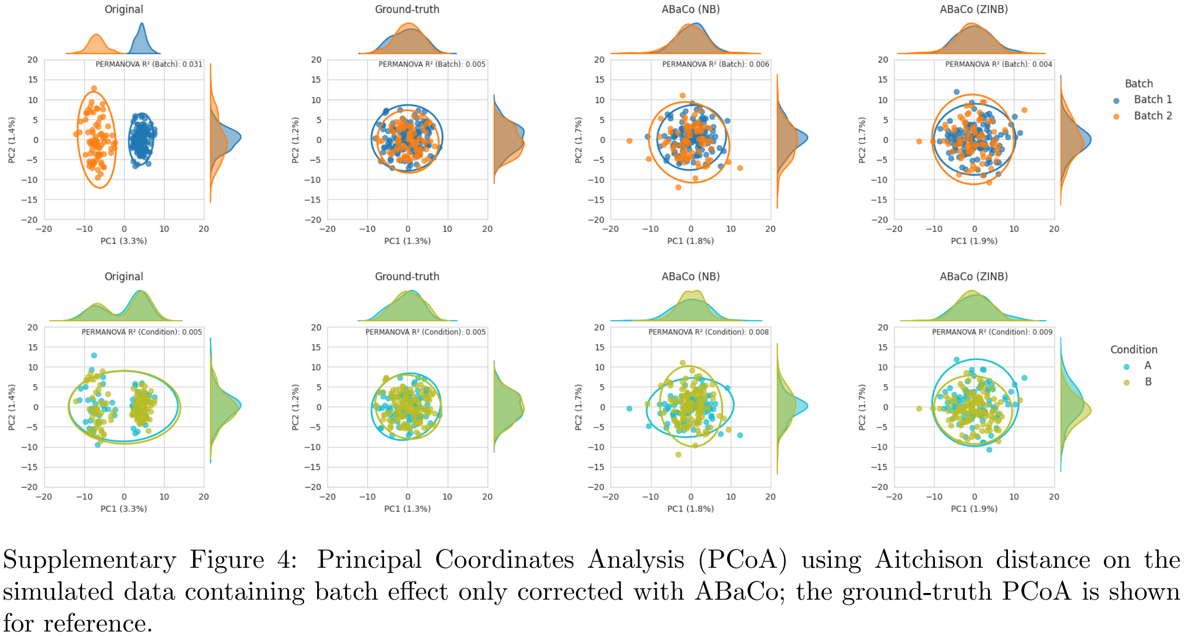

Supplementary Figure 4: Principal Coordinates Analysis (PCoA) using Aitchison distance on the simulated data containing batch effect only corrected with ABaCo; the ground-truth PCoA is shown for reference.

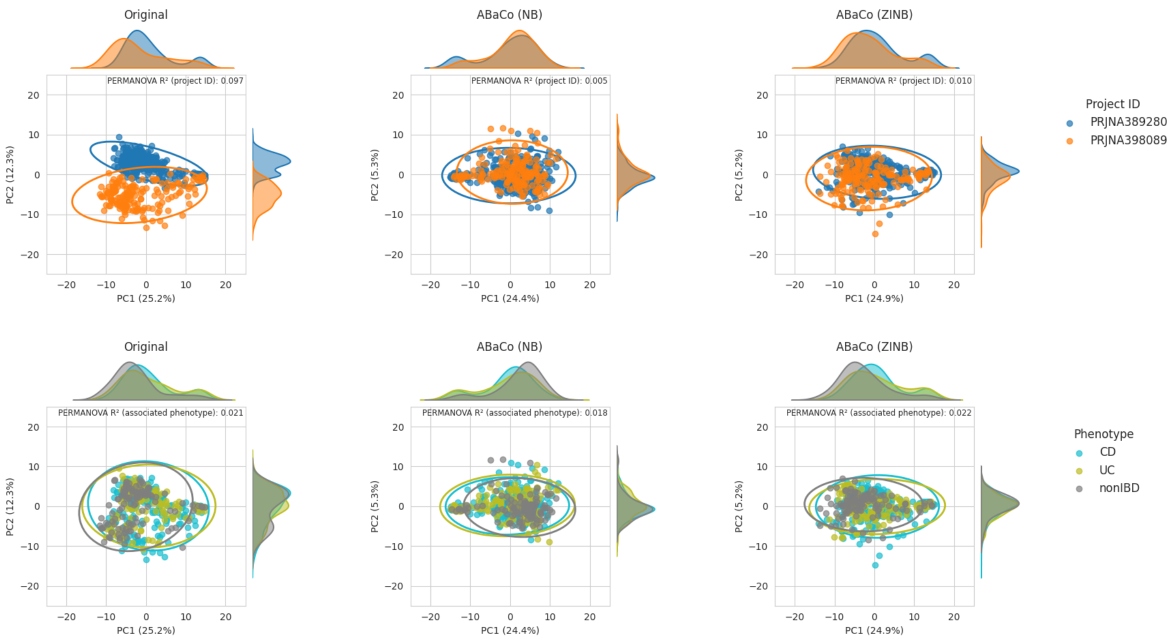

Supplementary Figure 5: Principal Coordinates Analysis (PCoA) using Aitchison distance on the IBD case study dataset corrected with ABaCo.

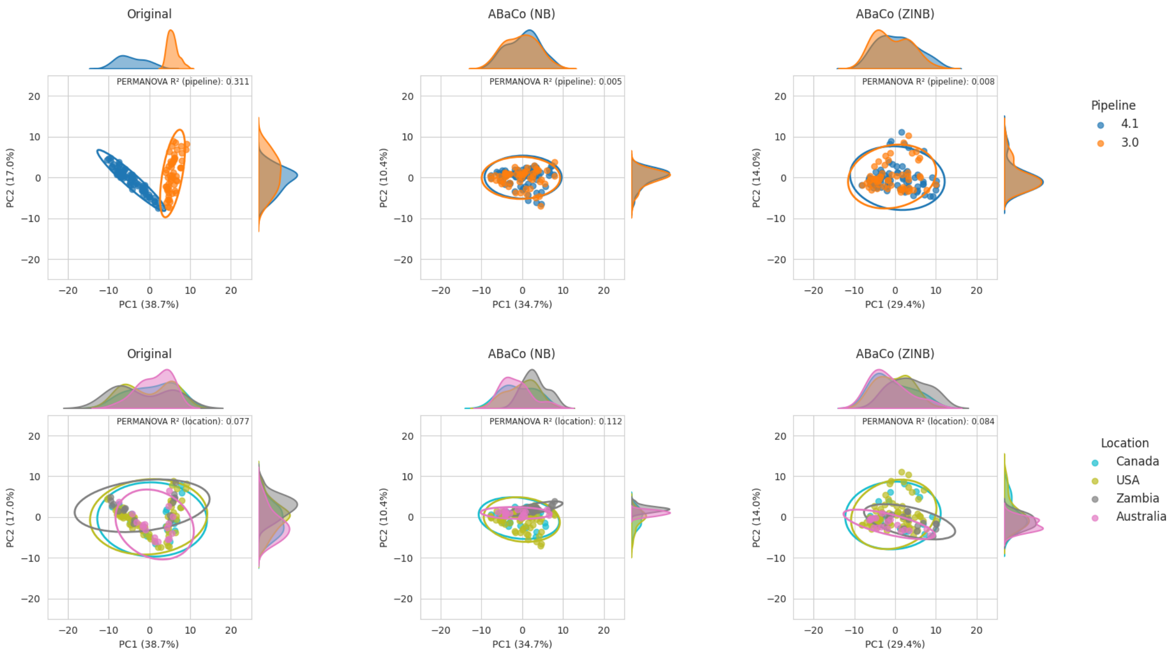

Supplementary Figure 6: Principal Coordinates Analysis (PCoA) using Aitchison distance on the DTU-GE sewage case study dataset corrected with ABaCo.

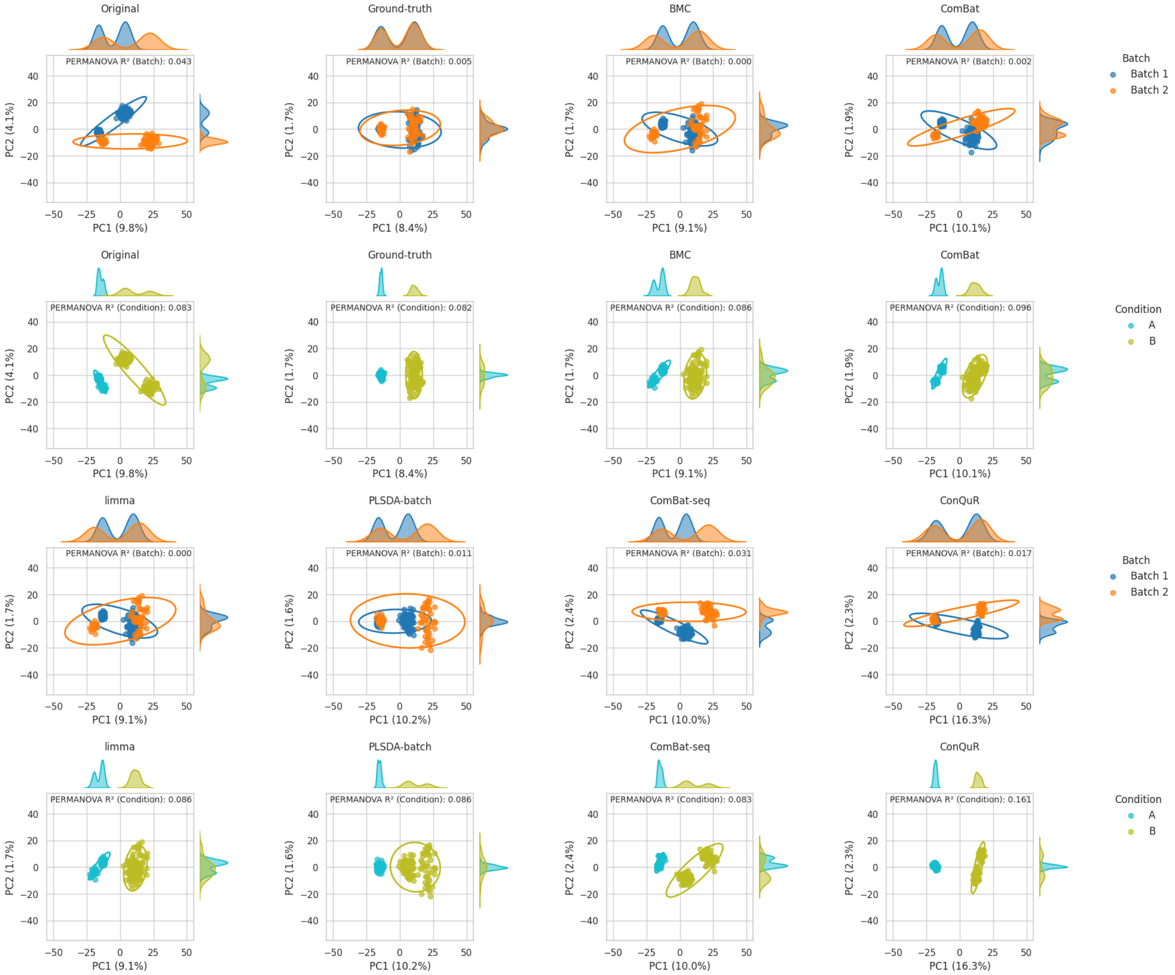

Supplementary Figure 7: Principal Coordinates Analysis (PCoA) using Aitchison distance on the simulated data containing both batch and biological effect corrected with state-of-the-art methods; the ground-truth PCoA is shown for reference.

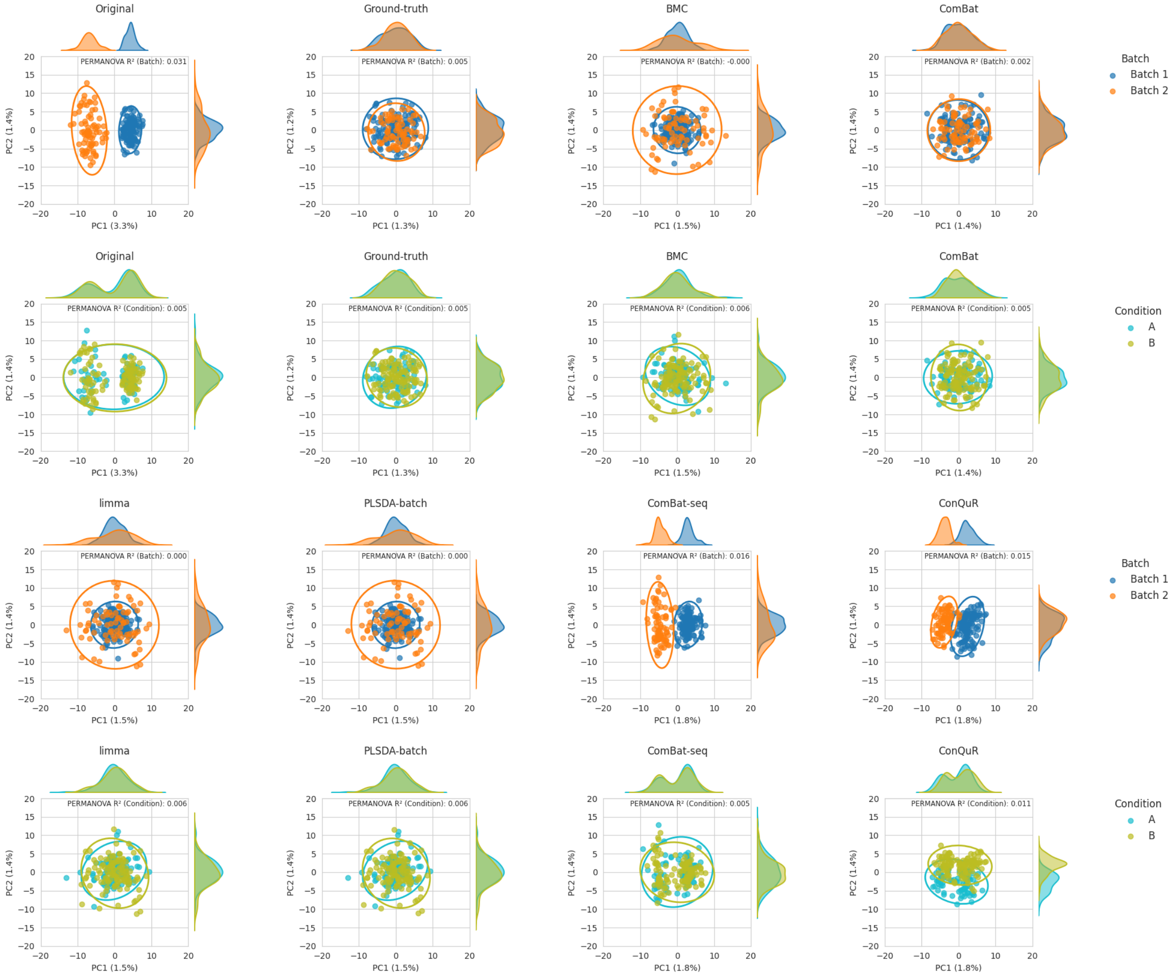

Supplementary Figure 8: Principal Coordinates Analysis (PCoA) using Aitchison distance on the simulated data containing batch effect only corrected with state-of-the-art methods; the ground-truth PCoA is shown for reference.

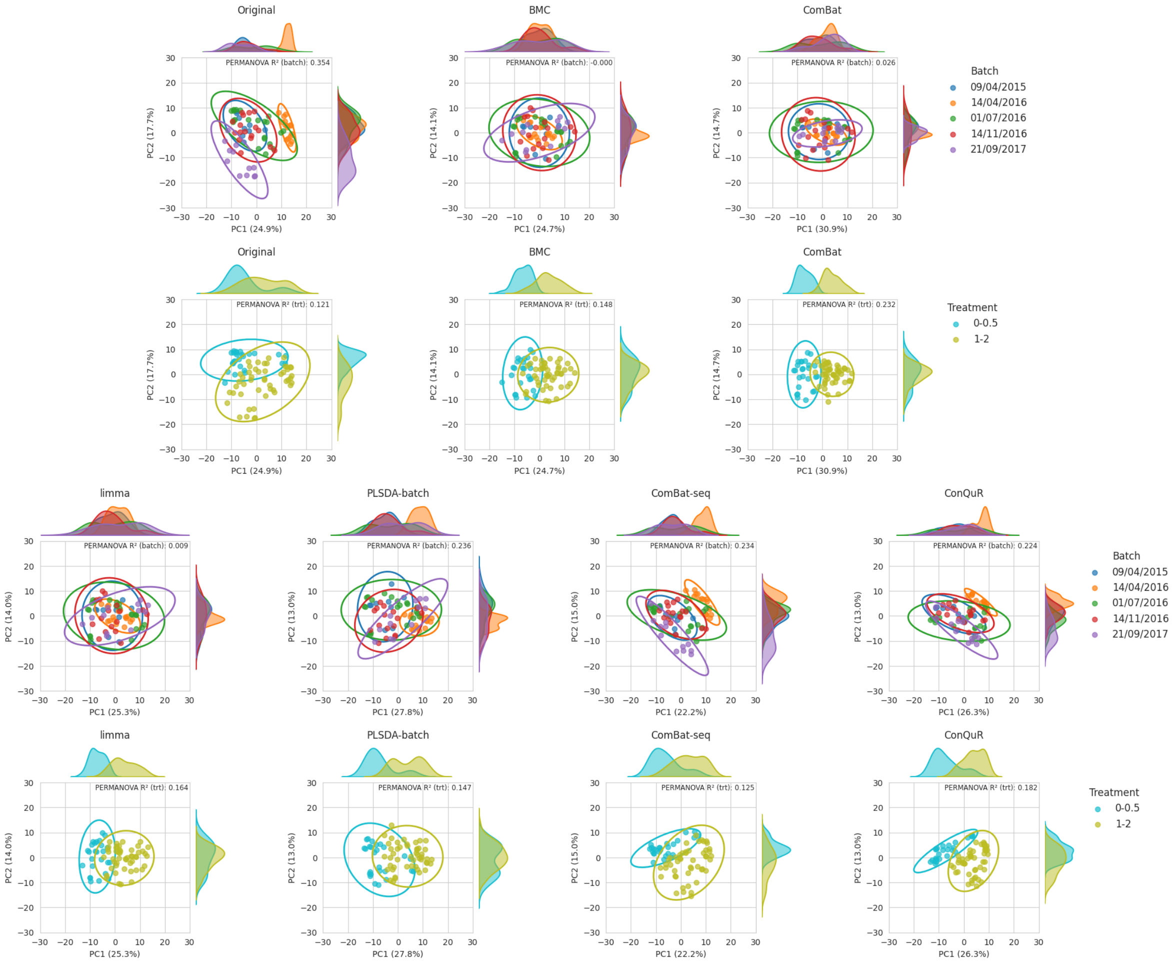

Supplementary Figure 9: Principal Coordinates Analysis (PCoA) using Aitchison distance on the anaerobic digestion case study dataset corrected with state-of-the-art methods.

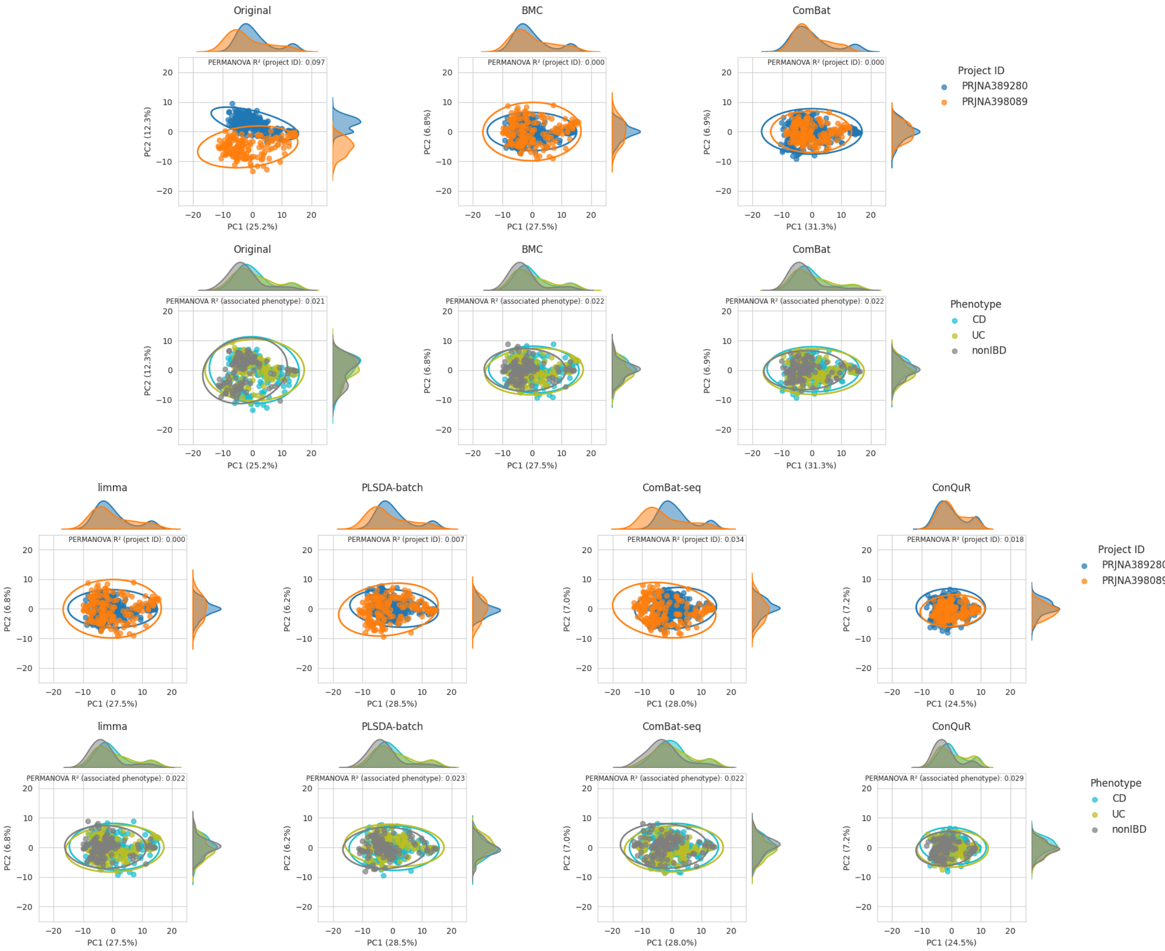

Supplementary Figure 10: Principal Coordinates Analysis (PCoA) using Aitchison distance on the IBD case study dataset corrected with state-of-the-art methods.

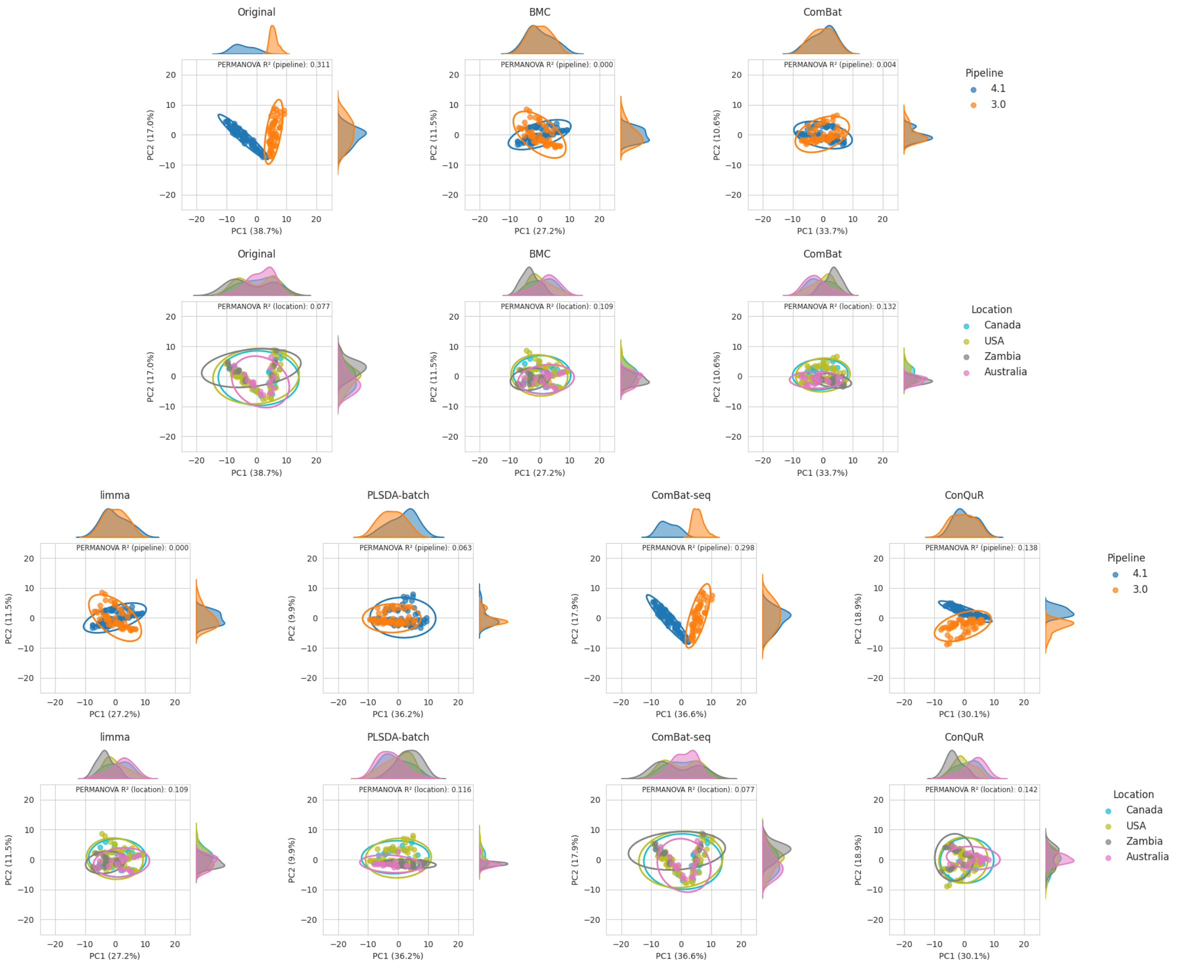

Supplementary Figure 11: Principal Coordinates Analysis (PCoA) using Aitchison distance on the DTU-GE sewage case study dataset corrected with state-of-the-art methods.

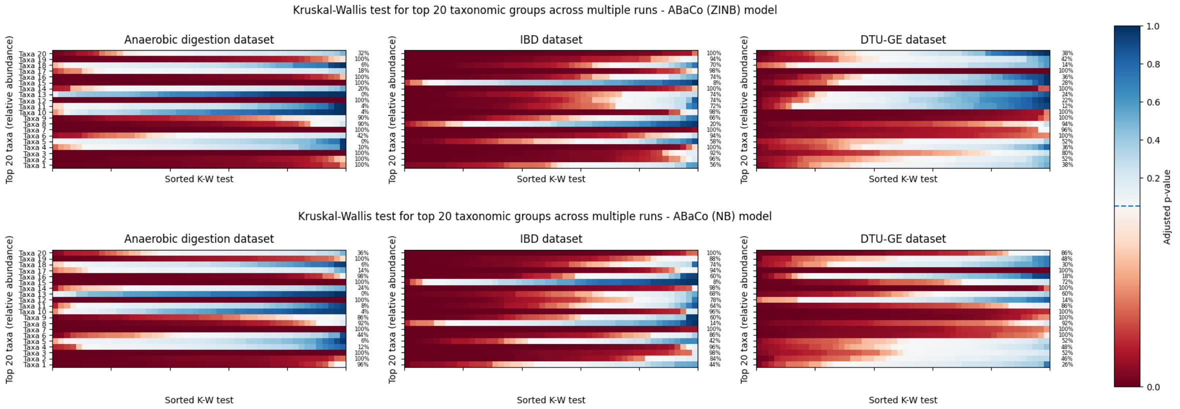

Supplementary Figure 12: Kruskal-Wallis test results in most abundant taxa for each case study dataset corrected with ABaCo.

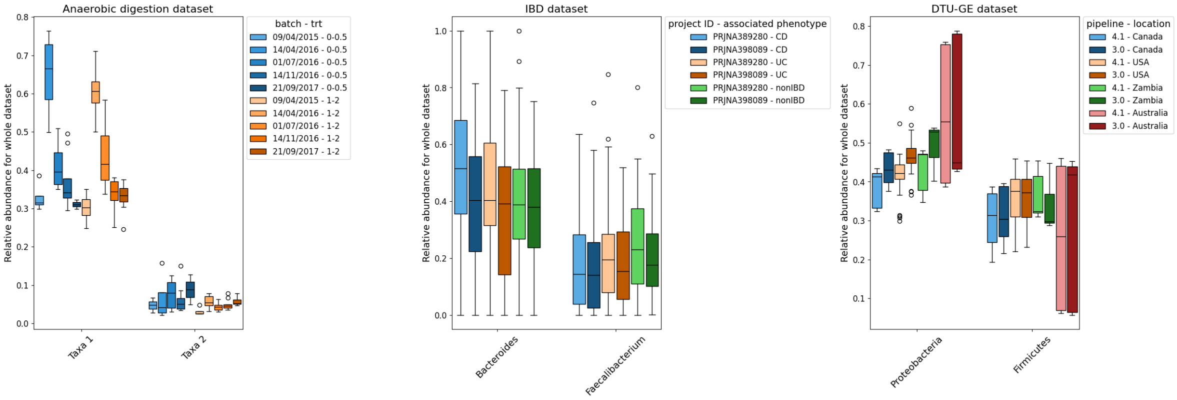

Supplementary Figure 13: Relative abundance of the two main taxa for all case study datasets stratified by biological and batch groups.

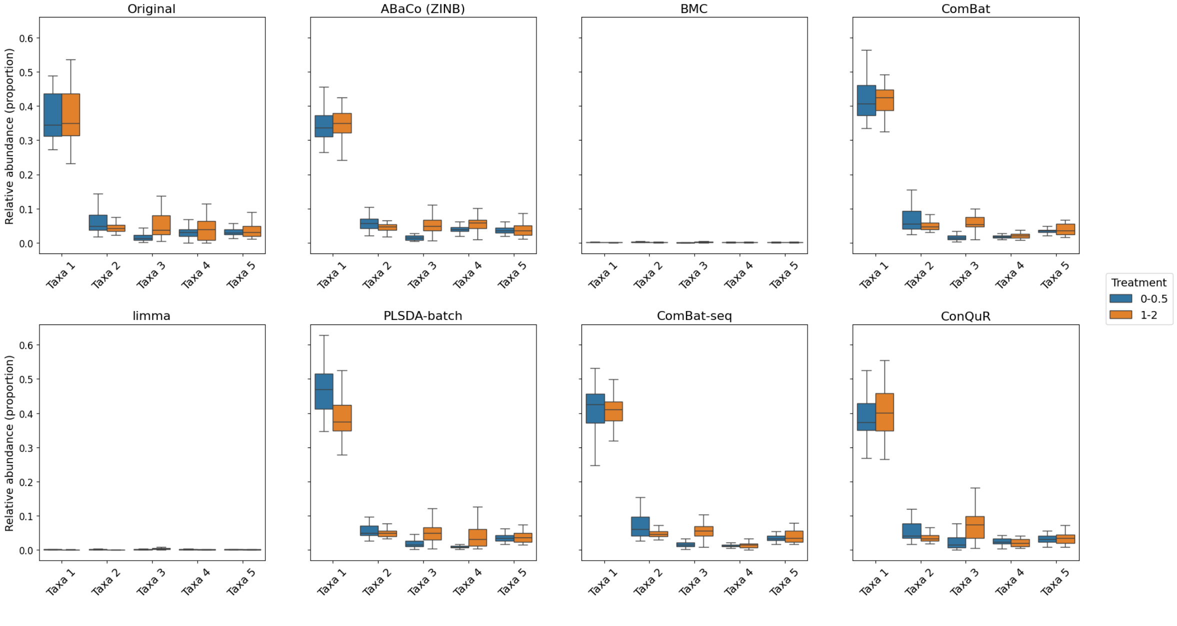

Supplementary Figure 14: Relative abundance of the five most abundant taxa for the anaerobic digestion case study dataset using every method (ABaCo random seed = 42).

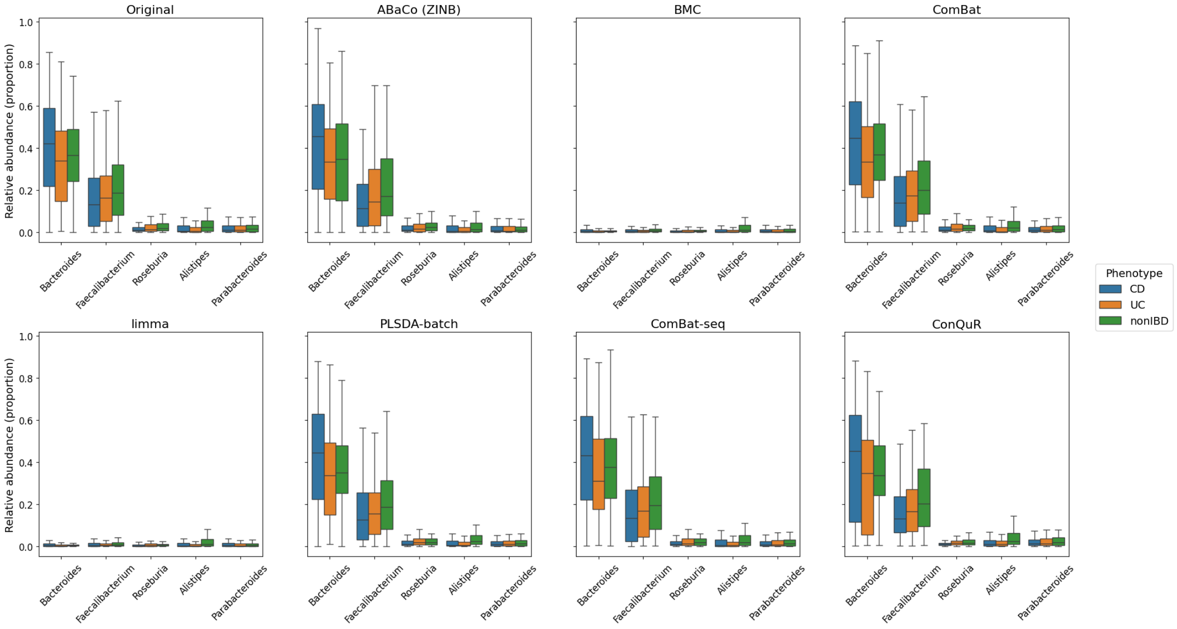

Supplementary Figure 15: Relative abundance of the five most abundant taxa for the inflammatory bowel disease case study dataset using every method (ABaCo random seed = 42).

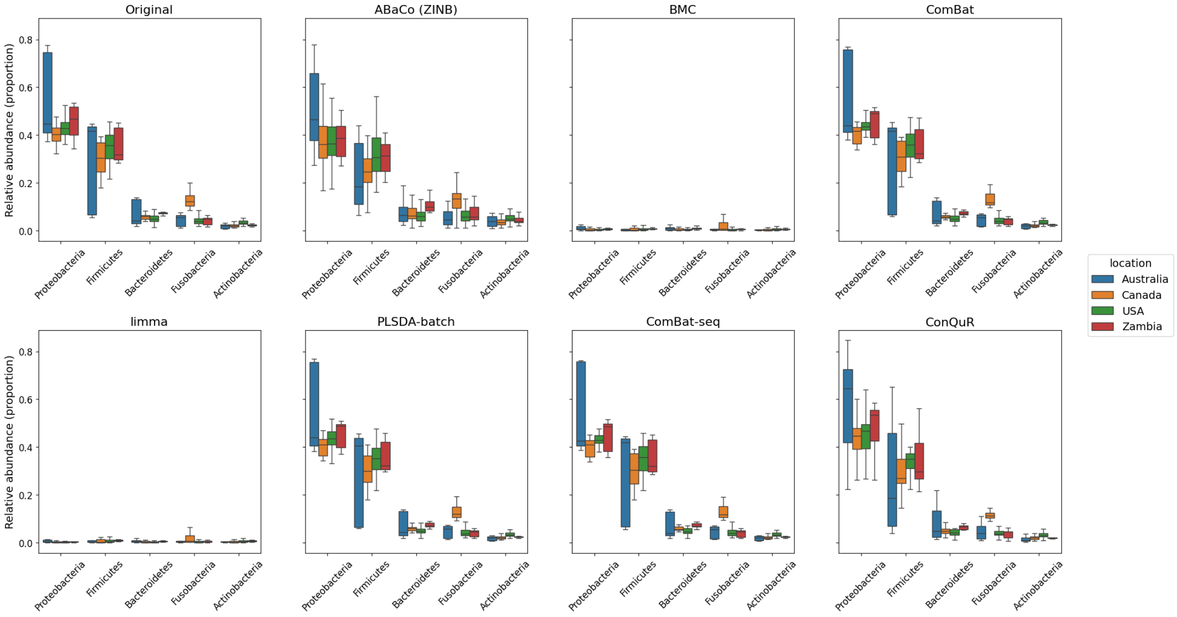

Supplementary Figure 16: Relative abundance of the five most abundant taxa for the DTU-GE sewage case study dataset using every method (ABaCo random seed = 42).

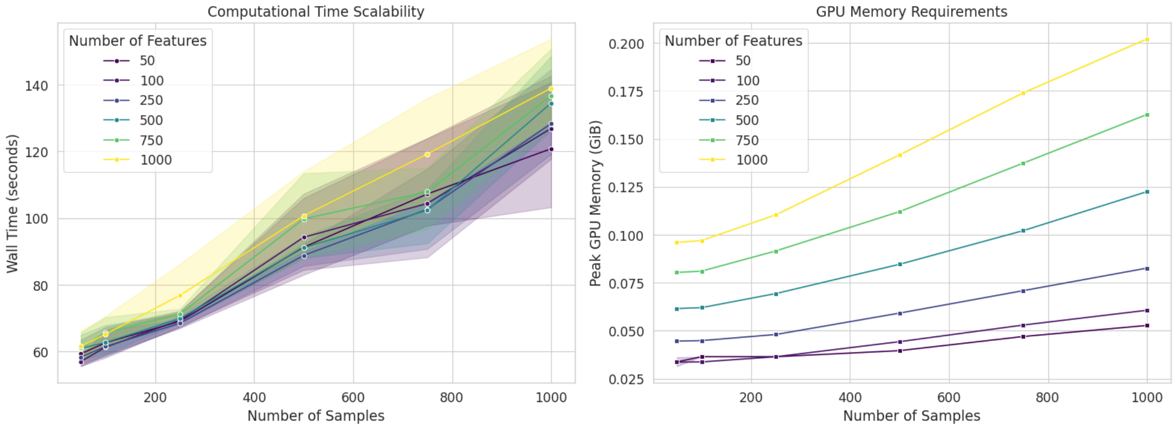

Supplementary Figure 17: Computational resources assessment of ABaCo. **Left**: Wall-clock training time (seconds) versus number of samples, stratified by number of features (colors). Solid lines represent the mean over 5 independent runs, while shaded regions denote the 95% confidence interval, illustrating training stability. **Right**: Peak GPU memory usage (GiB) versus number of samples. The model exhibits a highly efficient memory footprint, peaking at approximately 0.20 GiB for the largest dataset tested.
