# Supplementary material for: ABaCo: addressing heterogeneity challenges in metagenomic data integration with adversarial generative models
Source: Nucleic Acids Res. 2026 Mar 17;54(5):gkag227. doi: 10.1093/nar/gkag227 (PMC12993454; doi:10.1093/nar/gkag227)
Supplement: gkag227_Supplemental_Files [file gkag227_supplemental_files.zip › Supplementary_File_2_rev.pdf]

# Supplementary Material

## Theoretical Model Framework

In this supplementary material, we provide formal definitions and implementation details that complement the Methods section. We describe each of the component of the core ABaCo model (encoder, decoder and variational prior) and present explicit formulations of all loss terms. ABaCo extends the core variational autoencoder [1] by introducing a set of auxiliary objectives designed to structure the latent space into more organized and biologically meaningful representations.

## Encoder

Following the definition in [1], let $x \in \mathbb{R}^D$ denote the observed data and $z \in \mathbb{R}^d$ the latent variable, where $d \ll D$. The encoder is parameterized by $\phi$, and defines the approximate posterior distribution $q_\phi(z|x)$:

$$z \sim q_\phi(z|x) \tag{1}$$

In the ABaCo model, the encoder generates the parameters that define $K$ Gaussian components, $\{\mathcal{N}(\mu_{\phi,\mathrm{k}}(x), \sigma^2_{\phi,\mathrm{k}}(x))\}_{\mathrm{k}=1}^K$, along with the mixing probabilities $\pi(x)$ over these components. A component index $c$ is then sampled from the categorical distribution defined by $\pi(x)$, resulting in the approximate posterior:

$$q_\phi(z|x) = \mathcal{N}(\mu_{\phi,c}(x), \sigma^2_{\phi,c}(x)), \quad c \sim \mathrm{Categorical}(\pi(x)) \tag{2}$$

## Decoder

The decoder is parameterized by $\theta$, and defines the generative process for reconstructing counts from the latent variable $z$ as:

$$x \sim p_\theta(x|z) \tag{3}$$

In the baseline ABaCo model, the decoder employs a zero-inflated negative binomial (ZINB) distribution as the output layer, because it effectively captures both the sparsity and over-dispersion commonly observed in metagenomic count data. The decoder outputs the parameters of the ZINB distribution: the mean $\mu_\theta(z)$, the dispersion $\psi_\theta(z)$, and the zero-inflation probability $\pi_\theta(z)$. Accordingly, the conditional distribution of the observed data is given by:

$$p_\theta(x|z) = \mathrm{ZINB}(\mu_\theta(z), \psi_\theta(z), \pi_\theta(z)) \tag{4}$$

## Variational prior distribution

ABaCo employs the concept of the VampPrior Mixture Model (VMM) [2] to define the variational prior distribution. The variational prior distribution is parameterized by $\lambda$ with learnable parameters $\{\{\mathrm{u_k}\}, \{\sigma^2_\mathrm{k}\}\}_{\mathrm{k}=1}^K$. It is composed of a set of $K$ Gaussian components $\{\mathcal{N}(\mu_\mathrm{k}, \sigma^2_\mathrm{k})\}_{\mathrm{k}=1}^K$, where each component mean $\mu_\mathrm{k}$ is associated with a pseudo-input $\mathrm{u_k}$. To initialize the pseudo-inputs, we compute the arithmetic mean of all data points belonging to the same biological group. The variance parameters $\sigma^2_\mathrm{k}$ of each Gaussian component are initialized randomly, while the mean parameters $\mu_\mathrm{k}$ are obtained by passing the corresponding pseudo-input $\mathrm{u_k}$ through the encoder network:

$$\mu_\mathrm{k} = f_\phi(\mathrm{u_k}) \tag{5}$$

where $f_\phi(\mathrm{u_k})$ denotes the encoder mapping parameterized by $\phi$. Given a dataset $x = \{x_n\}_{n=1}^N$, the KL-divergence between the approximate posterior and prior distribution is not calculated against

the full set of prior components. Instead, we compute a component-wise divergence. Specifically, the component $c_n$ inferred for $x_n$ by the approximate posterior $q_\phi(z|x)$ is matched with the corresponding prior component, thereby preventing arbitrary label switching across components. The resulting KL-divergence for the observed data $x$ is therefore:

$$\mathrm{KL}\big(q_\phi(z|x)\,\|\,p_\lambda(z|\pi(x))\big) = \frac{1}{N}\sum_{n=1}^{N}\mathrm{KL}\big(\mathcal{N}(\mu_{\phi,c_n}(x_n), \sigma^2_{\phi,c_n}(x_n))\,\|\,\mathcal{N}(\mu_{c_n}, \sigma^2_{c_n})\big) \tag{6}$$

With this mechanism we state that our notation $p_\lambda(z|\pi(x))$ should be interpreted as the specific prior component selected based on the mixing probabilities of $x$.

## Biological group assignment loss

The main purpose of this loss is to ensure that the mixing probabilities $\pi(x)$ learned by the encoder are consistent with the true biological group assignment $c_{\text{bio}}(x)$. This encourages samples belonging to the same biological group to be assigned to the same latent component. The loss is thus defined as the categorical cross-entropy between the predicted mixing probabilities $\pi(x)$ and the one-hot encoded biological group labels $c_{\text{bio}}(x)$:

$$\mathrm{CE}(\pi(x), c_{\text{bio}}(x)) = -\sum_{k=1}^{K} c_{\text{bio},k}(x)\log \pi_k(x) \tag{7}$$

This loss acts as a supervised objective that anchors the probabilistic assignments to biologically meaningful partitions, guiding the latent space to better reflect known biological structure.

## Clustering regularizer loss

Given the variational prior distribution having multiple Gaussians, we employ a regularization term to incentivize separation between these components. This prevents the Gaussians from collapsing onto each other and ensures diversity in the latent space. The loss focuses on the two closest components (i.e. the pair most at risk of overlapping) and force them apart. The clustering regularizer is defined as the inverse of the minimum pairwise KL-divergence between every component of the prior distribution:

$$\mathcal{L}_{\text{cluster}}(\lambda) = \min_{i<j}\left(\sum_{h=1}^{d}\left[\log\frac{\sigma_{j,h}}{\sigma_{i,h}} + \frac{\sigma^2_{i,h} + (\mu_{i,h} - \mu_{j,h})^2}{2\sigma^2_{j,h}} - \frac{1}{2}\right]\right)^{-1} \tag{8}$$

where $d$ is the dimensionality of the latent space, and the inner sum ($\sum_{h=1}^{d}$) runs over all latent dimensions. Concretely, $\mu_{i,h}$ and $\sigma^2_{i,h}$ denote the mean and variance of prior component $i$ along latent dimension $h$. The expression therefore computes the univariate KL-divergence for each latent coordinate $h$ and sums those contributions to obtain the full divergence between the two Gaussians. The pair with the smallest divergence is selected, and by inverting this the loss creates a penalty that becomes negligible when groups are well-separated, but grows asymptotically large whenever any two groups are close to overlap. By enforcing separation of the prior components in the latent space, this regularizer encourages the emergence of distinct biological subgroups.

## Batch-mixing adversarial training

The adversarial training setup follows previous work in single-cell transcriptomics [3] [4]. This setup is employed to mitigate unwanted batch effects in the latent space. Given the batch discriminator logits over batch assignments $D(z)$ and the one-hot encoded batch labels $c_{\text{batch}}(z)$, the general adversarial min-max objective between the encoder $\mathcal{E}$ and the discriminator $D$ is:

$$\min_{D}\max_{\mathcal{E}}\; -\sum_{b=1}^{B} c_{\text{batch},b}(z)\log D_b(z) \tag{9}$$

In this setup, the discriminator $D$ tries to minimize the standard cross-entropy loss by correctly predicting the batch label, while the model encoder $\mathcal{E}$ tries to maximize the same loss to remove batch-specific information. Accordingly, the encoder's adversarial loss can be defined as:

$$\mathcal{L}_{\text{adv}}(D(z), c_{\text{batch}}(z)) = \sum_{b=1}^{B} c_{\text{batch},b}(z) \log D_b(z) \tag{10}$$

This corresponds to the negative cross-entropy between predicted and true batch labels. By optimizing this adversarial objective, the encoder learns to remove batch-specific signals, resulting in a latent representation that is more biologically meaningful and batch-invariant.

## References


[1] Tomczak, J. M. *Deep Generative Modeling* (Springer, 2024). URL https://link.springer.com/book/10.1007/978-3-031-64087-2.

[2] Stirn, A. A. & Knowles, D. A. The vampprior mixture model (2025). URL https://arxiv.org/abs/2402.04412. 2402.04412.

[3] Danino, R., Nachman, I. & Sharan, R. Batch correction of single-cell sequencing data via an autoencoder architecture. *Bioinformatics Advances* **4**, vbad186 (2023). URL https://doi.org/10.1093/bioadv/vbad186. https://academic.oup.com/bioinformaticsadvances/article-pdf/4/1/vbad186/55399346/vbad186.pdf.

[4] Shree, A., Pavan, M. K. & Zafar, H. scDREAMER for atlas-level integration of single-cell datasets using deep generative model paired with adversarial classifier. *Nat. Commun.* **14**, 7781 (2023).
